# In vitro comparison of the physical and mechanical properties of an ormocer with an ormocer‐based composite and a nanocomposite restorative material

**DOI:** 10.1002/cre2.756

**Published:** 2023-06-20

**Authors:** Karien Jansen van Rensburg, Dorette Kritzinger, Samantha Arnold, Glynn D. Buchanan

**Affiliations:** ^1^ Department of Odontology, School of Dentistry, Oral and Dental Hospital, Faculty of Health Sciences University of Pretoria Riviera South Africa; ^2^ Present address: Department of Operative Dentistry Sefako Makgatho Health Sciences University Ga‐Rankuwa South Africa

**Keywords:** microleakage, ormocers, surface hardness, surface roughness

## Abstract

**Objectives:**

To compare an ormocer with a first generation ormocer‐based composite and a nanocomposite in terms of surface roughness, surface hardness, and microleakage.

**Materials and Methods:**

An ormocer (Admira Fusion), a first generation ormocer‐based composite (Admira) and a nanocomposite (Filtek Z350 XT) were prepared strictly in accordance with the manufacturer's instruction and recommendation to provide optimal material properties. Twelve disk samples of each material were evaluated to assess both surface roughness and surface hardness. For surface roughness, all samples were finished, polished, and Ra values measured with a profilometer. For surface hardness, samples were stored in an incubator, polished and a Vickers diamond indenter was used to record values. For microleakage, 36 standardized, Class V cavities were prepared and randomly divided into three groups. Restored teeth were thermally fatigued, immersed in 2% methylene blue solution for 48 h, sectioned, and scored for occlusal and gingival microleakage.

**Results:**

Statistical significance was set at *p* < .05. The one‐way analysis of variance identified no significant difference in terms of surface roughness between the three material groups (*p* > .05). A significantly higher surface hardness was identified for the nanocomposite compared to both the ormocer (*p* < .001) and ormocer‐based composite (*p* < .001). Fisher's exact test identified no significant difference in terms of occlusal microleakage (*p* = .534) and gingival microleakage (*p* = .093) between the three material groups.

**Conclusions:**

No significant differences in terms of surface roughness or microleakage were noted. The nanocomposite was significantly harder than the ormocer materials.

## 1 INTRODUCTION

Resin composite is a popular, universally used, tooth colored, direct restorative material (Zimmerli et al., 2010). Interest in resin composite increased due to the material's superior esthetics and advantages of adhesive technology when used in conjunction with a bonding system (Zimmerli et al., 2010). Controlling the degree of cure, polymerization shrinkage and adhesion to adhesive systems and tooth structure are critical to improve biocompatibility and biofunctional properties of resin‐based dental composites (Alshali et al., 2013). Materials are exposed to stress during polymerization shrinkage of the matrix as well as aging in the oral environment (Ferracane et al., 1998). Saliva's aqueous medium, masticatory forces, variations in pH, and temperature fluctuations exert detrimental effects on the resin matrix and fillers (Monsarrat et al., 2017). Persistent complications, such as gap formation with the possibility of secondary caries, were experienced with initial tooth‐colored restorations, caused by polymerization shrinkage (Browning & Dennison, 1996). Additional complications included fractures due to the loss of occlusal relationships, increased degradation, and wear (Browning & Dennison, 1996). To overcome these obstacles, new matrices were developed and the filler content manipulated in terms of size, shape, type, and amount of filler used in the resin composite matrix (Monsarrat et al., 2017).

Much progress has been made regarding the composition of filler technology in resin composites over the years (Rosin et al., 2007). However, no fundamental changes have been made to the composition of the monomer matrix since the introduction of dimethacrylates by Bowen in the early 1960 (Bowen, 1962). In response to this, ormocers, a new dimethacrylate‐diluent‐free matrix formulation, were developed as a new material class (Moszner et al., 2008). The word ormocer is an acronym for organically modified ceramic (Wolter et al., 1992). This unique material group is an organically modified, non‐metallic, inorganic compound material (Wolter et al., 1992). Ormocers were originally designed for use in science and technology (Wolter et al., 1994), and were manufactured for special uses such as non‐stick surfaces, non‐reflective coatings, protective coatings, and anti‐static coatings (Zimmerli et al., 2010). Ormocers differ uniquely from conventional composites in that the matrix has both an organic and inorganic component (Wolter et al., 1994). Synthesis of the ormocer matrix is based on a saline precursor (Wolter et al., 1994). Multifunctional urethane and thioether(meth)acrylate alkoxysilanes are used to synthesize this material via a solution and gelation (sol‐gel) process (Wolter et al., 1994). Organically modified ceramic particles are created by hydrolyses and inorganic polycondensation in this sol‐gel process (VOCO, 2021). Unlike conventional composites that present with a carbon backbone, the ormocer resin matrix consists of an inorganic silicon dioxide backbone on which polymerizable carbon‐carbon double‐bond‐containing side‐chains are grafted, producing three‐dimensional compound polymers (Bottenberg et al., 2007). Ormocers can therefore be described as three dimensionally cross‐linked copolymers within the matrix presenting as polymers even before light curing (Kalra et al., 2012; Wolter et al., 1994).

The first generation of ormocers was expected to combine both the advantages of inorganic polymers (e.g., thermal stability, mechanical strength, and chemical resistance) and organic polymers (e.g., flexibility and impact resistance) (Shafqat et al., 2014). However, due to the ongoing challenges to improve handling properties and to incorporate filler particles, conventional dental monomers, such as bisphenol‐A glycidyl dimethacrylate (Bis‐GMA) and urethane dimethacrylate (UDMA), had to be added to the ormocer matrix, diminishing the initial promising advantages of this material (Moszner et al., 2008; Stadermann & Klemm, 1998). It therefore becomes necessary to refer to this added dimethacrylate, first generation ormocer materials as ormocer‐based composites (Moszner et al., 2008).

The ormocer, Admira Fusion (VOCO GmbH), was introduced to the market in 2015, as the world's first pure ceramic‐based restorative material (VOCO, 2016). Admira Fusion features pure ormocer matrix chemistry without the addition of conventional dimethacrylates (Moszner et al., 2008; VOCO, 2016). Ormocers are formed with the combination of nanohybrid technology and ormocer technology (Sivakumar & Valiathan, 2006; VOCO, 2021). This material is prominent because of its highly cross‐linked structure on the one hand and its tremendous amounts of linking units in the form of the double bonds on the other (Kalra et al., 2012; VOCO, 2021). It has a much higher bond compatibility than conventional composites because of the high degree of cross linkages between the chemical elements (VOCO, 2021).

Obtaining adequate surface hardness ensures that restorative materials will be able to withstand forces in dental stress bearing areas (Marghalani, 2010a). Surface roughness is an important property for clinical esthetics, resistance to dental plaque accumulation and ultimately the longevity of the restoration (Mair et al., 1996). Without an adequate marginal seal, microleakage will occur at the tooth‐restoration interface, resulting in failure of the restoration (Sudhapalli et al., 2018). Surface roughness, surface hardness, and microleakage can therefore be regarded as material properties that contribute to the longevity of resin composite restorations (Mair et al., 1996; Marghalani, 2010a; Sudhapalli et al., 2018). There is currently a limited body of evidence comparing the material properties of ormocers to ormocer‐based composites and nanocomposites.

The aim of this study was to determine whether a new generation of ormocers exhibits any clear advantages in terms of surface roughness, surface hardness, and microleakage when compared to conventional nanocomposites.

An additional aim of this study was to determine if there have been any improvements in pure ormocers compared to the first generation ormocer‐based composites in terms of surface roughness, surface hardness, and microleakage.

The null hypothesis was that there would be no difference in terms of surface hardness, surface roughness, and microleakage between the new generation ormocer, nanocomposite, and ormocer‐based composite.

## 2 MATERIALS AND METHODS

Ethical approval for this study was granted by the Research Ethics Committee, Faculty of Health Sciences, University of Pretoria (protocol number 207/2019). Composition, batch number and manufacturer's of the materials used in this study are listed in Table 1. Polishing systems and adhesives were used strictly in accordance with the recommendation and instruction of the individual manufacturer's of the restorative materials, to provide optimal material properties.

**Table 1:** Composition, batch numbers, and manufacturer's of all materials used in this study.

Material	Composition	Filler content, percentage	Manufacturer's	Batch number
Admira Fusion Nanohybrid ormocer	Matrix: Resin ormocer Filler: Silicon oxide nano filler, glass ceramics filler (1 µm)	84 (w/w) 69 (v/v)	VOCO GmbH	1915328 1945202
Futurabond U Bonding system	34% phosphoric acid Adhesive: Liquid 1: Acidic adhesive monomer HEMA BIS‐GMA, HEDMA, UDMA catalyst Liquid 2: Ethanol initiator, catalyst.		VOCO GmbH	1917239
Admira Microhybrid ormocer‐based composite	Matrix: Bis‐GMA, UDMA, TEGDMA Filler: Glass ceramic silicon oxide (0.7 µm)	79 (w/w) 56 (v/v)	VOCO GmbH	1908546
Admira Bond Bonding system	36% phosphoric acid Adhesive: Acetone, ormocer matrix, DMA polyfunctional methacrylate, CQ stabilizer		VOCO GmbH	1917136
Filtek Z350 XT Nanofilled composite other trade names for Filtek Z350 XT: Filtek Supreme XTE, Filtek Supreme Plus, Filtek Supreme Ultra, Filtek Supreme, Filtek supreme XT	Matrix: Bis‐GMA, TEGDMA, UDMA, Bis‐EMA Filler: Silica nanofillers (5−75 nm) zirconia/silica nanoclusters (0.6−1.4 µm)	78.5 (w/w) 59 (v/v)	3M, ESPE	NA62301
ESPE Adper Single Bond Universal Adhesive bonding system	36% phosphoric acid with colloidal silica Adhesive: Bis‐GMA, HEMA, DMA, polyalkenoic acid copolymer, initiator, water, ethanol		3M, ESPE	5695133

Abbreviations: Bis‐GMA, bisphenol‐A glycidyl dimethacrylate; UDMA, urethane dimethacrylate.

Data was observed on a continuous scale and groups were compared using analysis of variance (ANOVA) and when adjusting for baseline an analysis of covariance was used. Variation was expected to be small and hence for each of the three experiments a sample size of at least 12 samples per group, that is, at least 36 per experiment, were adequate as the residual degrees of freedom were at least 33. The latter is in line with the norm for acceptable sample size when residual degrees of freedom are at least 30. A conservative approach was followed here, compared to the norm of 14 residual degrees of freedom, which is often accepted when small variation is expected, that is, 6 teeth per group.

### 2.1 Sample fabrication

#### 2.1.1 Surface roughness and surface hardness

Twelve samples of each material, a ormocer (Admira Fusion; VOCO GmbH) a first generation ormocer‐based composite (Admira; VOCO GmbH) and a nanocomposite (Filtek Z350 XT; 3M; ESPE), were prepared using cylindrical aluminum moulds, 10 mm diameter x 2 mm height (Kritzinger et al., 2017). The same investigator performed all sample preparations. Shade A2 was chosen and a 2 mm material thickness since most composites are placed and cured in 2 mm increments (Caughman et al., 1995; Rueggeberg et al., 1993). Samples for both surface roughness and surface hardness were prepared in the same manner.

Each material was expressed into cylindrical molds using a composite gun. The moulds were slightly overfilled with each material and Mylar strips placed on either side of the uncured material and pressed between two glass slides, 1 mm thick, in accordance with previously described methodology (Beltrami et al., 2018; Kritzinger et al., 2017). Light finger pressure was used to extrude excess material (Beltrami et al., 2018; Kritzinger et al., 2017). Thereafter, all materials were polymerized, through a 1 mm glass slide; using a D‐Light Pro dual wavelength LED curing light (GC Europe) on a high power mode (1400 mW/cm^2^). Polymerization was performed through the top and bottom of the glass slide for the duration instructed by the individual manufacturer's (20 s for Amira Fusion, 40 s for Admira, and 10 s for Filtek Z350 XT) (Taher, 2005). A bluephase radiometer (Ivoclar Vivadent) was used to test the intensity of the curing light before curing each sample.

#### 2.1.2 Microleakage

A total of 18 non‐carious, crack and restoration free human premolars, extracted for reasons unrelated to this study, were collected in accordance with the ethical guidelines of The Research Ethics Committee, Faculty of Health Sciences, University of Pretoria. After debridement with a universal scaler (NSK Varios 370; NSK), the teeth were stored in distilled water at room temperature for no longer than 1 month (Haller et al., 1993; Hooshmand et al., 2013; Mahmoud & Al‐Wakeel Eel, 2011).

Using a high speed handpiece (NSK DynalLED M600LG QD; NSK) mounted with a water‐cooled, diamond dome end fissured bur (ISO 838.012 E11.001FG; Edenta AG), 36 box‐shaped, standardized, non‐bevelled Class V cavities were prepared on the buccal and lingual surface of each tooth (Haller et al., 1993; Hooshmand et al., 2013). Parameters of each cavity were approximately 3 mm x 3 mm x 2 mm, outlined by a permanent marker and dimensions confirmed using a Hu‐Friedy Williams periodontal probe (Hu‐Friedy Mfg. Co.; LLC) (Hooshmand et al., 2013; Jacker‐Guhr et al., 2016). The bur was replaced after every fifth preparation, and the cavities were prepared in such a way that the cemento‐enamel junction was located in the middle of each preparation (El‐Housseiny & Farsi, 2002; Hooshmand et al., 2013; Synarellis et al., 2017).

Following preparation, the teeth were randomly divided into three groups of six teeth containing a buccal and lingual cavity preparation. In each group, the cavities were packed with a restorative material and its corresponding bonding system according to the recommendations and instructions of the respective manufacturer's (Group 1: Admira Fusion with Futurabond U [VOCO GmbH], Group 2: Admira with Admira Bond [VOCO GmbH], and Group 3: Filtek Z350 XT with Adper Single Bond Universal Adhesive [3M ESPE]). Composition, batch number, and manufacturer's of all bonding systems are listed in Table 1. Using the recommended bonding agent for each of the restorative materials ensured optimal material properties for each individual material. The restorative materials were placed in a single increment and light cured using a D‐Light Pro dual wavelength LED curing light (GC Europe). A bluephase radiometer was used to test the intensity of the curing light before curing each sample. Restorations were then worked off with fine‐grit finishing flame shaped diamond burs and polished with the manufacturer's recommended polishing systems (Erdilek et al., 2009; Hooshmand et al., 2013). The same operator prepared all of the cavity preparations, as well as the total etch techniques, bonding, and material placement for each sample.

### 2.2 Surface roughness

After polymerization, the material samples were removed from the molds using finger pressure, and glued to a transparent plastic backing. Before polishing samples were mounted on a wheel template with three markings: 0°, 120,° and 240°. Three Ra measurements in different directions were recorded for each sample using a Surftest SJ 210 profilometer (Mitutoyo). This was done in accordance with previous studies to ensure a representative surface roughness value for the entire sample and not only the roughness of a certain area on the sample (Korkmaz et al., 2008; Kritzinger et al., 2017; Scheibe et al., 2009; Türkün & Türkün, 2004). The profilometer was calibrated after each sample.

Samples were then finished with a fine grit (red stripe), flame‐shaped finishing diamond bur ISO 806 314 249 514 012 (Dentsply Sirona/Maillefer) followed by an extra fine grit (yellow stripe) flame shaped finishing diamond bur, ISO 806 314 249 504 012 (Dentsply Sirona/Maillefer), under copious amounts of water spray for 10 s (Kritzinger et al., 2017). The rotation speed of the bur, 200,000 rpm, was regulated by an NSK NLX nano electric micromotor (NSK). Samples were finished and polished using the manufacturer's recommended polishing system (Dimanto; Voco GmbH for Admira and Amira Fusion and Sof‐Lex Diamond Polishing System; 3Mm ESPE for Filtek Z350 XT) in the direction of an arrow that was marked on a transparent plastic backing as performed in a previous study (Senawongse & Pongprueksa, 2007). A new polishing bur was used for each sample and polished for 20 s, under copious water‐cooling. After polishing, the samples were again mounted on transparent backing and three measurements of the polished surface in different directions were recorded for each sample.

### 2.3 Surface hardness

To simulate the oral environment, samples were placed in distilled water and stored in an incubator (Binder ED23) at 37 ± 1°C, for 24 h (Ciccone‐Nogueira et al., 2007; Poggio et al., 2018; Say et al., 2003). One surface of each sample was polished with silicon carbide paper in the series of 400‐800‐1200 grit under profuse water‐cooling (Hooshmand et al., 2013; Manhart et al., 2000; Say et al., 2003). Hardness was then tested using a Vickers Diamond Indenter (Struers; Duramin‐40 AC 3) at a 500 g load with a dwell time of 40 s (Hahnel et al., 2010; Say et al., 2003). Five hardness values were recorded on the polished surface of each sample and averaged as a single value (Poggio et al., 2018; Schneider et al., 2011). The five indentations were equally placed in a straight line and neither closer than 0.5 mm to the adjacent indentation (Poggio et al., 2018).

### 2.4 Microleakage

Following preparation, samples were stored in distilled water within a Binder incubator at 37 ± 1°C, for 7 days to allow for water‐absorption and to simulate the oral environment (Erdilek et al., 2009; Hooshmand et al., 2013; Poggio et al., 2018; Say et al., 2003). After storage, each group was marked and subjected to 3000 cycles of thermocycling (Cooling and heat bath: PolyScience; Thermal cycler: Proto‐tech) varying between 5°C and 55°C, with a dwell time of 20 s to simulate clinical stress (Hooshmand et al., 2013; Synarellis et al., 2017). Silicon moulds where then used to seal the root apices with clear self‐cure acrylic resin (Excel Rapid Repair Cold Cure Acrylic; Wright Health Group Ltd), to prevent dye penetration through the apical foramen (Sudhapalli et al., 2018). Tooth surfaces were coated with two coats of nail varnish up to 2 mm from the margins of each restoration (Hooshmand et al., 2013; Kubo et al., 2001). Samples were immersed in 2% methylene blue dye solution for 48 h at room temperature, after which they were rinsed and dried (Kalra et al., 2012). The nail varnish was removed with hand instruments and the samples were embedded in clear self‐cure acrylic resin, using a silicone mold (Erdilek et al., 2009). An ISOMET low‐speed precision section blade (Buehler) with water‐cooling was used to cut each sample vertically in a bucco‐lingual direction through the center of the restoration (Hooshmand et al., 2013; Kubo et al., 2001). Using an Olympus SZX7 Stereomicroscope (Olympus Corporation) at x25 magnification, sections of each group were visually examined for dye penetration (Erdilek et al., 2009; McHugh et al., 2017).

**Figure 1:**
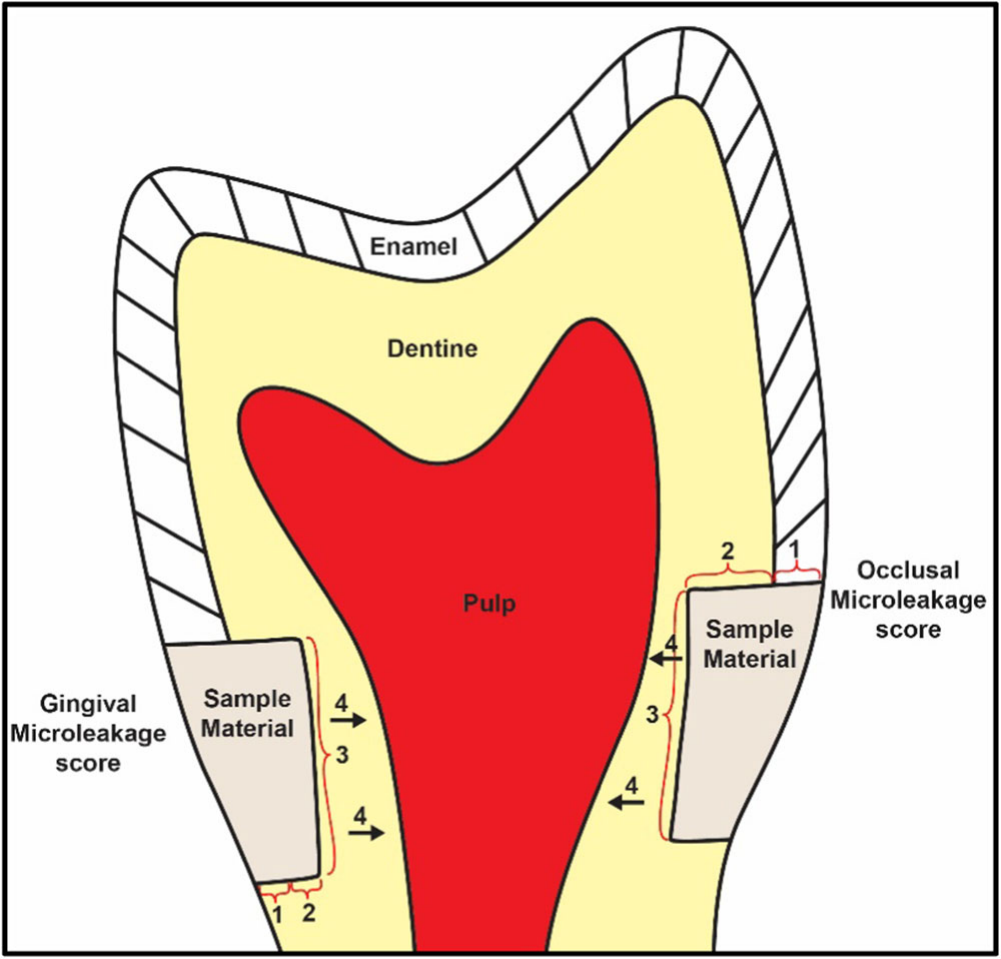
Graphical illustration of the criteria used to score the occlusal and gingival microleakage.

Microleakage was scored according to the criteria outlined in Figure 1, described as follows:

Occlusal margin: (Garapati et al., 2014)

1‐ No dye penetration.
2‐ Dye penetration not extending beyond the dentine‐enamel junction.
3‐ Dye penetration further than the dentine‐enamel junction but not beyond the junction of the occlusal and axial wall.
4‐ Dye penetration along the axial wall.
5‐ Dye penetration beyond the cavity depth in the pulpal direction.

Gingival margin: (Erdilek et al., 2009)

1‐ No dye penetration.
2‐ Dye penetration that extended less than or up to ½ of gingival wall.
3‐ Dye penetration further than ½ or up to ¾ of the gingival wall.
4‐ Dye penetration greater than ¾ of the gingival wall or up to and along the axial wall.
5‐ Dye penetration beyond the gingival and axial wall in the pulpal direction.

#### 2.4.1 Stereomicroscope images

**Figure 2:**
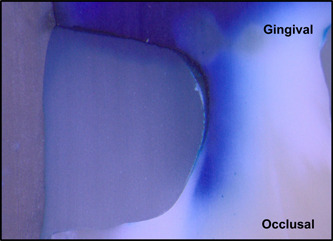
Sample with an occlusal microleakage score of 1 and a gingival microleakage score of 4.

**Figure 3:**
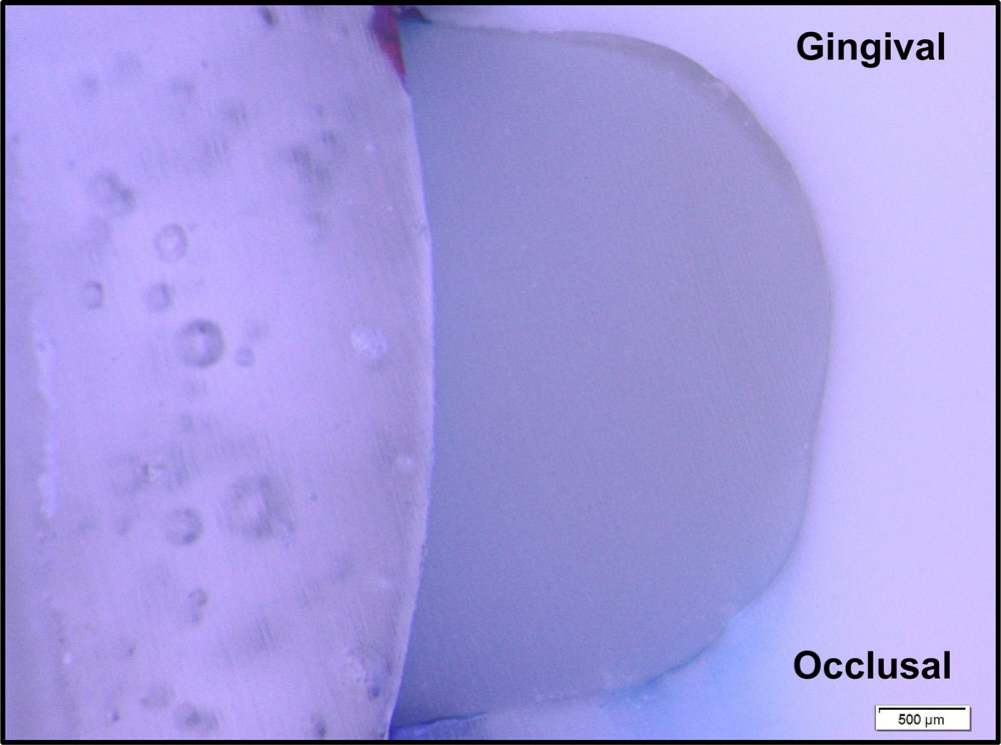
Sample with an occlusal microleakage score of 3 and a gingival microleakage score of 1.

**Figure 4:**
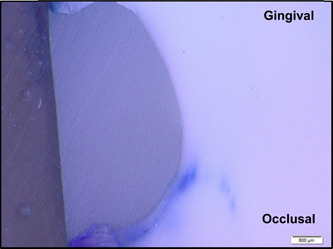
Sample with an occlusal microleakage score of 4 and a gingival microleakage score of 2.

**Figure 5:**
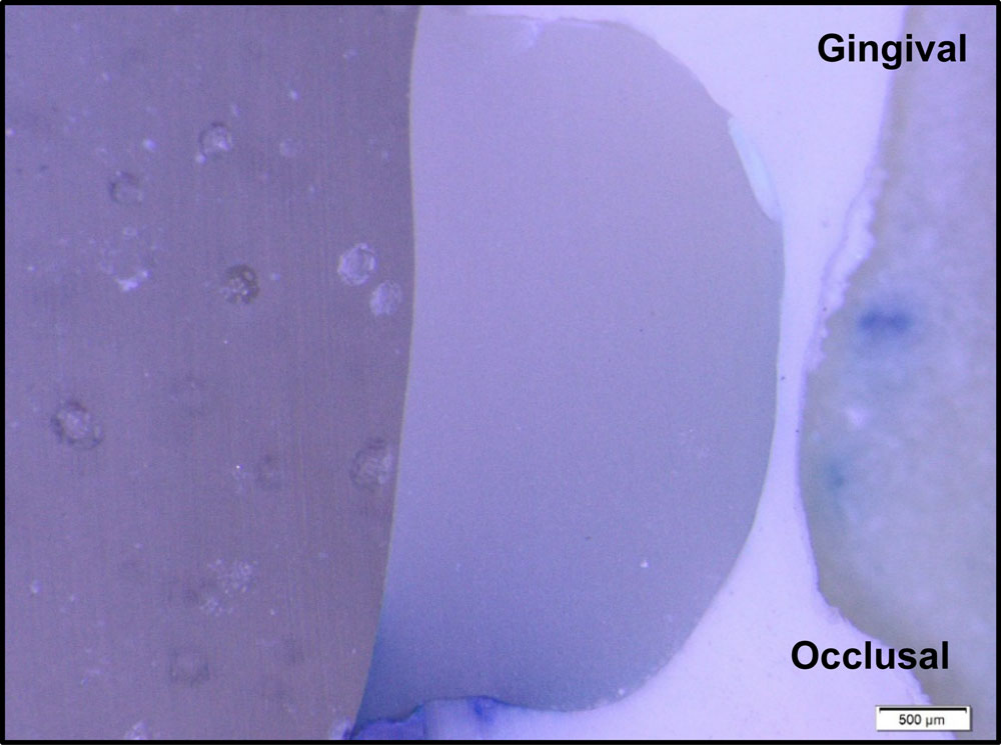
Sample with an occlusal microleakage score of 2 and a gingival microleakage score of 0.

**Figure 6:**
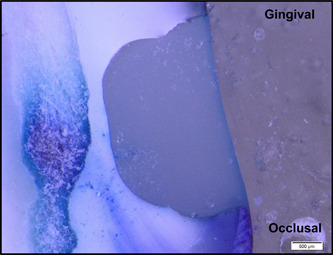
Sample with an occlusal microleakage score of 4 and a gingival microleakage score of 3.

Figures 2, 3, 4, 5, 6 are representative photos of each group of microleakage scoring (magnification x25).

The results of both surface roughness and surface hardness were compared in one‐way ANOVA. Occlusal and gingival microleakage data for the three materials were compared using Fisher's exact test. Pairwise comparisons among the materials were tested with the Bonferroni adjustment. Significant values were set at *p* < .05.

## 3 RESULTS

### 3.1 Surface roughness

No statistical significant differences were found between the three material groups (*p* > .05), before polishing. Pairwise comparisons between materials after polishing with the manufacturer's recommended polishing systems demonstrated no statistically significant difference between the surface roughness of the ormocer, with both the ormocer‐based composite and the nanocomposite (*p* > .05).

### 3.2 Surface hardness

Pairwise comparisons between the three tested materials differed significantly with respect to surface hardness (*p* < .05). The results of this study revealed that the ormocer and the ormocer‐based composite did not differ significantly (*p* = .617). The nanocomposite did however differ significantly from the ormocer (*p* < .001), showing statistically significant higher surface hardness vallues.

### 3.3 Microleakage

The results of the microleakage testing are demonstrated in Tables 2 and 3. No statistically significant differences were revealed in terms of occlusal microleakage between the ormocer with the ormocer‐based composite and the nanocomposite (*p* = .534) (Table 2).

**Table 2:** Occlusal microleakage scores.

Occlusal microleakage (%)						
Score	0	1	2	3	4	Total
Ormocer	8.31 (a1)	8.33 (a1)	16.67 (a2)	16.67 (a2)	50.00 (a6)	100 (a12)
Ormocer‐based composite	16.67 (a2)	16.67 (a2)	8.33 (a1)	41.67 (a5)	16.67 (a2)	100 (a12)
Nanocomposite	0	8.33 (a1)	16.67 (a2)	50.00 (a6)	25.00 (a3)	100 (a12)
	*p* b = .534.					

^a^ Number of the 12 samples tested with the same score.

^b^ Fisher's exact test.

**Table 3:** Gingival microleakage scores.

Gingival microleakage (%)						
Score	0	1	2	3	4	Total
Admira Fusion	0	0	0	33.33 (a4)	66.67 (a8)	100 (a12)
Admira	0	16.67 (a2)	8.33 (a1)	25.00 (a3)	50.00 (a6)	100 (a12)
Filtek Z350 XT	16.67 (a2)	16.67 (a2)	0	0	66.67 (a8)	100 (a12)
	*p* b = .093					

^a^ Number of the 12 samples tested with the same score.

^b^ Fisher's exact test.

Fisher's exact test also revealed no statistically significant differences between the materials with respect to gingival microleakage (*p* = .093) (Table 3).

## 4 DISCUSSION

In terms of surface roughness and microleakage, the null hypothesis was proven. For these material properties, no statistical differences between the ormocer, the ormocer‐based composite, or the nanocomposite existed.

However, in terms of surface hardness the null hypothesis was rejected. The nanocomposite showed statistical significantly higher surface hardness compared to the ormocer and the ormocer‐based composite.

### 4.1 Surface roughness

While the initial high shine of a restoration may be important to the patient, the surface quality of the restoration after months and years of service is of concern to the dentist (Ferracane, 2011).

Both intrinsic and extrinsic factors affect the surface roughness of resin composites (Marghalani, 2010b). Intrinsic factors include durability of the filler and resin matrix bond, filler type, size, shape and distribution, the type of material, its resin matrix composition, and degree of polymerization (Marghalani, 2010b). The various methods of finishing and polishing relate to the extrinsic factors and entail the characteristics of the polishing tool such as its flexibility, geometrical shape, abrasive particles, and its method of application (Buchgraber et al., 2011). In the current study, no statistically significant differences were found between the three material groups after curing through a Mylar strip before polishing. During the process of finishing and polishing, resin matrix is removed between the filler particles and as a result filler particles are more prominent on the composite surface, resulting in increased surface roughness (Goncalves et al., 2008). Finishing and contouring of the restoration after placement become necessary to correct the morphology and shape of a tooth before polishing (Anusavice & Phillips, 2003). To mimic the most likely clinical conditions, all samples were first contoured with diamond‐finishing burs. Previous studies have suggested that each material behaves independently of the various polishing techniques (Antonson et al., 2011; Marghalani, 2010b). Since certain polishing techniques are better suited to specific materials, polishing of the different materials in the current study was done strictly according to the manufacturer's recommendations and instructions.

After polishing each of the materials, the results of this study showed no statistically significant difference between the surface roughness of the ormocer with the ormocer‐based composite and the nanocomposite. The lack of significant differences suggest that the ormocer exhibits approximately the same surface roughness as the other two materials. This finding was similar to a study done by Hahnel et al. (2010), that showed no significant difference in the surface roughness of a ormocer when compared to a nanocomposite. In a study done by Baseren (2004), both the ormocer‐based composite (Admira) and the nanocomposite (Filtek Supreme; 3M ESPE) were polished with multiple different polishing systems, but there was no significant difference in surface roughness among the tested materials (*p* > .05). In Baserens' (2004) study there were, however, statistical significant differences between the different polishing systems applied to the materials. Cunha et al. (2003) compared the surface roughness of two ormocer‐based composites with that of a microhybrid conventional composite after tooth brushing and found no significant differences between these materials.

### 4.2 Surface hardness

Hardness is a surface characteristic that is defined as the ability of a material to resist permanent indentation or penetration (Yap et al., 2002). Both the type and amount of filler in composite materials greatly influence its surface hardness (Schmage et al., 2009). Mechanical properties, such as polishability and abrasion resistance, are greatly dependant on material hardness (Schmage et al., 2009). It has also been shown that surface hardness can act as an indicator of the degree of polymerization of a material (Asmussen, 1982). The higher the conversion rate of carbon double bonds, the better the physical properties and surface hardness of a material (Manhart et al., 2000).

In the current study storage for 24 h at 37°C allowed for the continued setting reaction during “dark polymerization” (Pilo & Cardash, 1992) and a further increase in surface hardness as the materials aged at body temperature (Marghalani, 2010a). After polymerization and storage, silicon carbide paper was used to remove the soft resin‐rich layer of uncured monomers (Marghalani, 2010a). Removal of this layer allowed for testing of a stable harder surface and simulated clinical conditions since most restorations are polished after placement (Marghalani, 2010a).

The results of the current study showed no significant difference between the surface hardness of the ormocer (Admira Fusion) and the ormocer‐based composite (Admira). The lack of significant difference between these two materials suggests that in terms of surface hardness there may be no difference between using either of these materials. In a study done by Leprince et al. (2010), all samples were dried and stored in the dark for 24 h. The results of their study were in contrast to the current study as they found the ormocer to have increased surface hardness when compared to the ormocer‐based composites (Leprince et al., 2010). These findings can partly be explained by the higher filler content of an ormocer (Leprince et al., 2010). Cavalcante et al. (2011) also contradicted the findings of the current study. Their study compared the surface hardness of an ormocer to that of an ormocer‐based composite, nanohybrid composite and nanocomposite after 24 h water storage. The study concluded that the ormocer preserved surface integrity and was the only material that did not show a reduction in surface hardness values (Cavalcante et al., 2011). Water may diffuse internally through defects, pores, and the resin matrix, causing hydrolytic breakdown within the resin composite (Kalachandra & Wilson, 1992), which may explain the differences regarding these findings. The absence of methacrylate monomers in the pure ormocer matrix has been shown to reduce water uptake and solubility (Cavalcante et al., 2011). Ormocers present as polymers and tend to absorb water to a different degree (Mortier et al., 2005). During polymerization a chain reaction is initiated which enables the double bonds of each element of the matrix to react with one another to form a chain or a network respectively (VOCO, 2021). A three‐dimensional network of the organic portion of the methacrylate groups formed after polymerization (Kalra et al., 2012; Zimmerli et al., 2010). The unique matrix formulation of ormocers is therefore important and can explain the improved stability of this material compared to ormocer‐based composites and conventional composite materials (Cavalcante et al., 2011). The current study did revealed a statistical significant difference in surface hardness when the nanocomposite (Filtek Z350 XT) was compared with the ormocer (Admira Fusion). The findings of the current study correlate with the findings of Poggio et al. (2018), where a nanocomposite (Filtek Supreme XTE; 3M ESPE) showed the highest microhardness values followed by an ormocer (Admira Fusion) both before and after immersion in an acidic drink for a week. Baeshen et al. (2017). also found higher hardness values when a nanocomposite (Filtek Supreme XT; 3M ESPE) was compared to an ormocer (Admira Fusion) before surface finishing and polishing. A possible reason for this finding could be the materials' filler composition (Baeshen et al., 2017). Admira has barium glass fillers that are lower in hardness compared to the zirconia fillers used in Filtek Supreme nanocomposite restorative materials (Baeshen et al., 2017). The type of filler, filler load, and the interactions between the filler and matrix influence the surface hardness to a greater extent than the organic matrix structure (Manhart et al., 2000). The findings of the current study however contradict those of a study done by Hahnel et al. (2010), where the ormocer demostrated significantly higher Vickers hardness number value than the nanocomposite Filtek Supreme XT.

### 4.3 Microleakage

Marginal adaptation of a material to the cavity walls will determine the material's durability and longevity in the oral cavity (Sudhapalli et al., 2018). The main cause of microleakage is shrinkage that occurs in resin‐based materials during polymerization (Sudhapalli et al., 2018). The amount of polymerization shrinkage within a resin containing dental material is dependent on the filler load, the surface treatment of the fillers, and the molecular weight of the monomer (Sudhapalli et al., 2018). The more filler particles present in the material, the lower the amount of weak polymer matrix, the higher the strength and modulus of elasticity and the lower the polymerization shrinkage (Sudhapalli et al., 2018). Newer generation dental resin materials such as ormocers and nanocomposites show less polymerization shrinkage (Sudhapalli et al., 2018). Due to the nano‐sized particles of nanocomposites, less polymerization shrinkage and lower microleakage were observed, making this class of materials superior to conventional resin composites (Saunders, 2009). Ormocers also showed reduced polymerization shrinkage due to the highly cross‐linked, three‐dimensional polymer network that forms after polymerization (Kalra et al., 2012; Zimmerli et al., 2010).

Restorative materials tend to expand and contract more with temperature changes than enamel and dentine (Gonzalez et al., 1997; Nelsen et al., 1952). Thermocycling, with temperatures varying between 5°C and 55°C, was therefore used in the current study to mimic these thermal changes and stresses of the oral environment (Synarellis et al., 2017). No standardization has been established for the technical procedures of in vitro microleakage evaluations (Raskin et al., 2001). Of all the various microleakage test techniques available, the most common method used is dye penetration (Raskin et al., 2001; Sudhapalli et al., 2018). The present study ensured that each material was used with its proprietary adhesive system as recommended by the individual manufacturer's.

In the current study, no statistically significant differences were found in the occlusal microleakage of any of the three material groups. This would suggest that in terms of microleakage on enamel surfaces there may be no difference to using any of the tested materials. Politi et al. (2018) and McHugh et al. (2017) found significantly lower microleakage scores for the ormocer (Admira Fusion) when compared to a nanohybrid composite (Tetric EvoCeram; Ivoclar Vivadent). Garapati et al. (2014) found no significant difference in terms of microleakage between the ormocer‐based composite (Admira) and a nanocomposite (Filtek Supreme). Sudhapalli et al. (2018), compared microleakage between an ormocer‐based composite (Admira), nanocomposite (Tetric N‐Ceram; Ivoclar Vivadent), and a conventional microfilled composite (Tetric Ceram; Ivoclar Vivadent) with all margins ending on enamel. Their findings indicated that the ormocer‐based composite showed the least microleakage followed by the nanocomposite (Sudhapalli et al., 2018). Kalra et al. (2012) also found no significant difference in the microleakage of an ormocer‐based composite (Admira) compared to that of a hybrid composite (Spectrum TPH; Dentsply Sirona). The differences in the findings of the above‐mentioned studies may be attributed to the different materials compared and varying study methodologies.

Comparison of gingival microleakage revealed a marginally significant difference between the nanocomposite (Filtek Z350 XT; 3M ESPE) and the ormocer (Admira Fusion), with the nanocomposite showing marginally less leakage. No significant differences were found in the gingival microleakage when comparing the ormocer (Admira Fusion) to the ormocer‐based composite (Admira). Hooshmand et al. (2013) demonstrated significantly higher gingival microleakage for the ormocer‐based composite than the nanohybrid. A study done by Civelek et al. (2003), showed less microleakage at the cemento‐enamel junction for the ormocer‐based composite (Admira) when compared to a hybrid composite (Filtek Z‐250; 3M ESPE).

Material properties are not the only factors that need to be taken into account in the success or failure of a restoration. Other factors such as the adhesive force between the composite and the dentine, diameter and direction of the dentine tubules, as well as the quality and origin of the tooth's hard tissues, should also be taken into account (Leloup et al., 1998).

## 5 LIMITATIONS OF THE STUDY

The findings of in vitro studies are effective for the comparison of different materials and properties, however may not necessarily reflect the clinical situation. The present findings should therefore be interpreted with caution when extrapolated to the clinical environment. Each material and its bonding system was used strictly according to the recommendation of the individual manufacturer's to extract optimal material properties. Different results may have been found for microleakage if the same bonding system had been used for all materials. Future clinical studies may be beneficial to further compare these materials.

## 6 CONCLUSION

No significant differences in terms of surface roughness or microleakage were noted for the ormocer when compared to a commercially available nanocomposite as well as a first generation ormocer‐based composite. Nanocomposite demonstrated increased surface hardness compared to both ormocer materials. Long‐term clinical studies are necessary to assess the performance of this new ormocer material.

## AUTHOR CONTRIBUTIONS

**Dr. Karien Jansen van Rensburg**: Have made a substantial contribution to the conception, design, and acquisition of data as well as the analysis and interpretation of data. Drafted the manuscript and gave final approval for the version to be published. Agree to be accountable for all aspects of the work in ensuring that questions related to the accuracy or integrity of any part of the work are appropriately investigated and resolved. **Dr. Dorette Kritzinger**: Involved in data acquisition and analysis as well as interpretation of data. Revised the manuscript critically and gave final approval of the version to be published. **Dr. Samantha Arnold**: Revised the manuscript critically and gave final approval of the version to be published. **Dr. Glynn D. Buchanan**: Revised the manuscript critically and gave final approval of the version to be published.

## CONFLICT OF INTEREST STATEMENT

The authors declare no conflict of interest.

## Data Availability

Data is available upon request.
